# Synthesis and Biological Evaluation of Novel Benzothiazole Derivatives as Potential Anticonvulsant Agents

**DOI:** 10.3390/molecules21030164

**Published:** 2016-02-29

**Authors:** Da-Chuan Liu, Hong-Jian Zhang, Chun-Mei Jin, Zhe-Shan Quan

**Affiliations:** Key Laboratory of Natural Resources and Functional Molecules of the Changbai Mountain, Affiliated Ministry of Education, College of Pharmacy, Yanbian University, Yanji 133002, China; 2013001048@ybu.edu.cn (D.-C.L.); zhjzhishixiang@163.com (H.-J.Z.)

**Keywords:** synthesis, benzothiazole, mercapto-triazole, anticonvulsant, maximal electroshock, neurotoxicity, pentylenetetrazole

## Abstract

New benztriazoles with a mercapto-triazole and other heterocycle substituents were synthesized and evaluated for their anticonvulsant activity and neurotoxicity by using the maximal electroshock (MES), subcutaneous pentylenetetrazole (scPTZ), and rotarod neurotoxicity (TOX) tests. Among the compounds studied, compound 2-((1*H*-1,2,4-triazol-3-yl)thio)-*N*-(6-((3-fluorobenzyl)oxy)benzo[*d*]thiazol-2-yl)acetamide (**5i**) and 2-((1*H*-1,2,4-triazol-3-yl)thio)-*N*-(6-((4-fluorobenzyl)oxy)benzo[*d*] thiazol-2-yl)acetmide (**5j**) were the most potent, with an ED_50_ value of 50.8 mg/kg and 54.8 mg/kg in the MES test and 76.0 mg/kg and 52.8 mg/kg in the scPTZ seizures test, respectively. They also showed lower neurotoxicity and, therefore a higher protective index. In particular, compound **5j** showed high protective index (PI) values of 8.96 in the MES test and 9.30 in the scPTZ test, which were better than those of the standard drugs used as positive controls in this study.

## 1. Introduction

February ninth, 2015 was the first International Epilepsy Day, and it helped more people raise awareness and understanding about epilepsy. Such efforts are urgently needed, because with more than 50 million people presenting with epilepsy worldwide, epilepsy is the most common, chronic, serious neurological disease. In 2013, 119,000 deaths worldwide were attributable to epilepsy. Currently, 40% of patients in high-income countries and more than 70% of patients in developing countries do not get the treatment they need, because of the high expense or low availability of the appropriate drugs [1,2,3]. Therefore, there is a pressing need to develop more effective antiepileptic drugs (AEDs) endowed with an improved safety profile.

**Figure 1:**
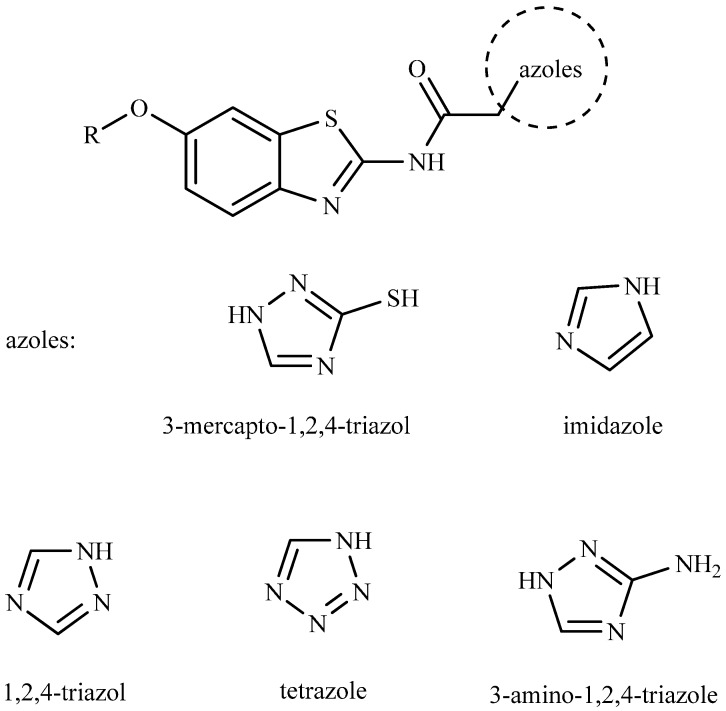
Benzothiazole and kinds of azoles combined with amide.

On the basis of a number of related materials, azoles and their derivatives have gained much attention in recent years due to their potential biological applications linked to their anticonvulsant [4], anti-inflammatory [5], anti-fungal [6], antiviral [7], and anticancer [8] activities. Among them, heterocyclic compounds with the 3-mercapto-1,2,4-triazole substructure exhibit a wide spectrum of biological activities [9,10]. Similarly, we also demonstrated that the benzothiazole nucleus is a unique scaffold for further molecular exploration to synthesize novel compounds. A literature survey revealed that benzothiazole analogs are associated with diverse pharmacological effects [11,12,13,14], including anticonvulsant activity [15,16]. For this reason, and in continuation to our efforts directed toward the synthesis of new heterocyclic compounds with anticonvulsant biological activities, in this study, we combined both biological components (3-mercapto-1,2,4-triazole and benzothiazole) with an amide, which has anticonvulsant bioactivities in some AEDs (such as carbamazepine and riluzole, also see references [17,18]), to obtain a series of 2-((1*H*-1,2,4-triazol-3-yl)thio)-*N*-(6-alkoxybenzo[*d*]thiazol-2-yl)acetamide. To compare the compound activities with those of other azoles, the mercapto-triazole in compound **5** was replaced with other heterocycles such as imidazole, triazole, tetrazole, and 3-amino-1,2,4-triazole, to obtain compounds **6**, **7**, **8**, and **9** (Figure 1). Their anticonvulsant activities were evaluated using the maximal electroshock (MES) test and their neurotoxicity (TOX) was evaluated with the rotarod test in mice.

## 2. Results and Discussion

### 2.1. Chemistry

**Scheme 1:**
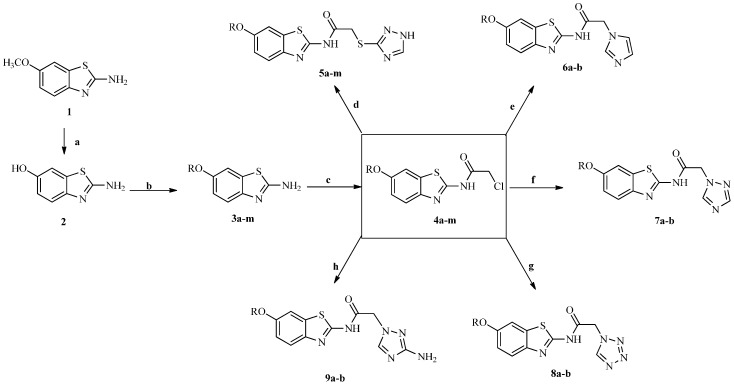
Synthetic route of target compounds. *Reagents and conditions*: (**a**) HBr (48 wt % in H_2_O, 99.99% metals basis), reflux, 18 h; (**b**) RBr/RPHCH_2_Cl, CH_3_COCH_3_, K_2_CO_3_, reflux, 18–24 h; (**c**) ClCOCH_2_Cl, acetone, room temperature, 9–10 h; (**d**–**h**) DMF, NaOH, room temperature, 8–12 h.

All the target compounds were synthesized according to Scheme 1. Compounds **3a**–**m** were prepared according to previous studies in our laboratory [19]. Compounds **3a**–**m** were treated with chloroacetyl chloride at room temperature in acetone to yield derivatives **4a**–**m** [20,21]. Finally, when derivatives **4a**–**m** were allowed to react with different azoles, such as 1*N*-1,2,4-triazole-3-thiol, imidazole, triazole, tetrazole, and 3-amino-1,2,4-triazole in refluxing dimethylformamide (DMF) in the presence of NaOH [22,23,24,25], the 2-chlorine atom was substituted by these heterocycles, producing the corresponding compounds: 2-((1*H*-1,2,4-triazol-3-yl)thio)-*N*-(6-alkoxybenzo[*d*]thiazol-2-yl)acetamide (**5a**–**m**), *N*-(6-alkoxybenzo[*d*]thiazol-2-yl)-2-(1*H*-imidazol-1-yl)acetamide (**6a**–**b**), *N*-(6-alkoxybenzo[*d*]thiazol-2-yl)-2-(1*H*-1,2,4-triazol-1-yl)acetamide (**7a**–**b**), *N*-(6-alkoxybenzo[*d*]thiazol-2-yl) -2-(1*H*-tetrazol-1-yl)acetamide (**8a**–**b**), and 2-(3-amino-1*H*-1,2,4-triazol-1-yl)-*N*-(6-alkoxybenzo[*d*] thiazol-2-yl)acetamide (**9a**–**b**). The structures of the targeted compounds were characterized using spectral methods, and all spectral data corroborated the assumed structures.

### 2.2. Pharmacology and Structure-Activity Relationship

The anticonvulsant activity evaluation of compounds 2-((1*H*-1,2,4-triazol-3-yl) thio)-*N*-(6-alkoxybenzo[*d*]thiazol-2-yl)acetamide (**5a**–**m**) were determined using the MES test, which is a mechanism-independent animal seizure model that enables the identification of compounds preventing seizure spread [26]. It should be noted that the MES model remains the most useful tool for the identification of new anticonvulsants, despite significant advances in epilepsy research in the past several years [27]. The MES seizure model was used for preliminary (phase I) screening of compounds **5a**–**m**. They were administered to mice intraperitoneally (i.p.) at the fixed dose of 100 mg/kg and the anticonvulsant protection was observed at two post-treatment times: 0.5 and 4 h. The method applied here allowed the determination of the number of animals (in a group consisting of three mice) protected against electrically-induced seizures as well as the estimation of the time course of anticonvulsant activity, including quick-acting (0.5 h) or long-acting properties (4 h). The results are presented in Table 1. The preliminary pharmacological screening revealed that five compounds (**5b**, **5c**, **5g**, **5i,** and **5j**) showed 100% anticonvulsant protection in the 0.5 h period and some of them (**5c**, **5i,** and **5j**) still had a little activity in the 4 h period. One compound, **5h**, showed 67% anticonvulsant protection in the 0.5 h period, but no activity in the 4 h period. None of the compounds presented neurotoxicity at the dose of 100 mg/kg. Based on the above preliminary data, six active compounds were screened at the dose of 30 mg/kg in mice (i.p.) at the two post-treatment times (0.5 h and 4 h). As shown in Table 2, only two compounds, **5i** and **5j**, showed about 33% anticonvulsant protection activity in the 0.5 h period but no activity in the 4 h period.

The following structure-activity relationships (SAR) were obtained, while analyzing the preliminary screening of the synthesized compounds. Among the six alkyl chain-substituted derivatives, **5b** and **5c** showed better activities, and **5c** still presented some activities in the 4 h period. However, with the increase in length, the activities of the compounds did not increase. Compound **5g,** substituted with a benzyl group at the 6-position of the benzothiazole core, showed moderate activity at 100 mg/kg. Thus, the F, Cl, and CF_3_ groups were subsequently added onto the benzyloxy group of **5g** at different positions, yielding compounds **5h**–**m**. Substituent position on the phenyl ring also influenced anticonvulsant activity in the 6-fluorobenzyl derivatives as *m*-F = *p*-F > *o*-F. However, the 6-chlorobenzyl derivatives showed no activity at the dose of 100 mg/kg. Compound **5m**, substituted with a trifluoromethyl at the 3-position of the benzyl group, also showed no activity, regardless of the period (0.5 h or 4 h) at the dose of 100 mg/kg. Based on the above-mentioned results, six compounds (**5b**, **5c**, **5g**, **5h**, **5i,** and **5j**) were selected from all the compounds for the next step and were tested at a dose of 30 mg/kg. As shown in Table 2, at the dose of 30 mg/kg, only *m*-F and *p*-F substituted compounds presented some anticonvulsant activities in the 0.5 h period.

**Table 1:** Anticonvulsant activities screening (maximal electroshock test) and neurotoxicity screening in mice at the dose of 100 mg/kg. 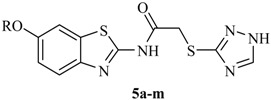

Comp.	R	MES (100 mg/kg) ^a^		Toxicity (100 mg/kg)	
		0.5 h	4 h	0.5 h	4 h
**5a**	-CH_3_	1/3 ^b^	0/3	0/3	0/3
**5b**	*n*-C_3_H_7_	3/3	0/3	0/3	0/3
**5c**	*n*-C_4_H_9_	3/3	1/3	0/3	0/3
**5d**	*n*-C_5_H_11_	0/3	0/3	0/3	0/3
**5e**	*n*-C_6_H_13_	1/3	0/3	0/3	0/3
**5f**	*n*-C_7_H_15_	1/3	0/3	0/3	0/3
**5g**	-CH_2_C_6_H_5_	3/3	0/3	0/3	0/3
**5h**	-CH_2_C_6_H_4_ (*o*-F)	2/3	0/3	0/3	0/3
**5i**	-CH_2_C_6_H_4_ (*m*-F)	3/3	1/3	0/3	0/3
**5j**	-CH_2_C_6_H_4_ (*p*-F)	3/3	1/3	0/3	0/3
**5k**	-CH_2_C_6_H_4_ (*o*-Cl)	0/3	0/3	0/3	0/3
**5l**	-CH_2_C_6_H_4_ (*m*-Cl)	0/3	0/3	0/3	0/3
**5m**	-CH_2_C_6_H_4_ (*m*-CF_3_)	0/3	0/3	0/3	0/3

^a^ Maximal electroshock (MES): doses of 100 mg/kg were administrated intraperitoneally in mice. The animals were examined at two times: 0.5 h and 4 h after administration; ^b^ n1/n2: the animals protected/the animals tested.

**Table 2:** Anticonvulsant activities screening: MES test in mice at the dose of 30 mg/kg.

Comp.	R	MES (30 mg/kg) ^a^	
		0.5 h	4 h
**5b**	*n*-C_3_H_7_	0/3	0/3
**5c**	*n*-C_4_H_9_	0/3	0/3
**5g**	-CH_2_C_6_H_5_	0/3	0/3
**5h**	-CH_2_C_6_H_4_ (*o*-F)	0/3	0/3
**5i**	-CH_2_C_6_H_4_ (*m*-F)	1/3	0/3
**5j**	-CH_2_C_6_H_4_ (*p*-F)	1/3	0/3

^a^ Maximal electroshock (MES): doses of 30 mg/kg were administrated intraperitoneally in mice.

According to the bioisosterism, the mercapto-triazole ring of compounds **5i** and **5j** was replaced with other heterocycles, such as imidazole, triazole, tetrazole, and 3-amino-1,2,4-triazole. Compounds **6**, **7**, **8**, and **9** were designed and synthesized. Their anticonvulsant activities were evaluated at the dose of 100 mg/kg, and screening results are shown in Table 3. The compounds, substituted with other heterocycles, hardly showed any anticonvulsant activities in the 0.5 h or 4 h period. Thus, we concluded that when the mercapto-triazole ring in compounds **5a**–**m** was replaced by other heterocycles (*i.e.*, imidazole, triazole, tetrazole, and 3-amino-1,2,4-triazole), the resultant compounds, **6a**–**b**, **7a**–**b**, **8a**–**b**, and **9a**–**b**, hardly presented any activities compared with the compounds containing the mercapto-triazole ring.

**Table 3:** Anticonvulsant activities of compounds **6**, **7**, **8**, and **9** in MES test. 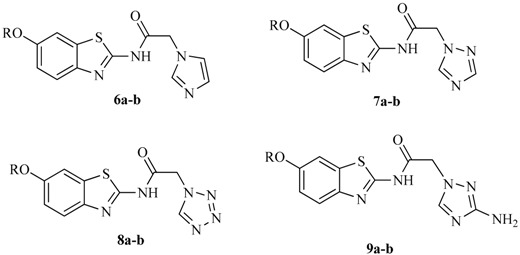

Comp.	R	MES (100 mg/kg)		Toxicity (100 mg/kg)	
		0.5 h	4 h	0.5 h	4 h
**6a**	-CH_2_C_6_H_4_ (*m*-F)	0/3	0/3	0/3	0/3
**6b**	-CH_2_C_6_H_4_ (*p*-F)	0/3	0/3	0/3	0/3
**7a**	-CH_2_C_6_H_4_ (*m*-F)	0/3	0/3	0/3	0/3
**7b**	-CH_2_C_6_H_4_ (*p*-F)	0/3	0/3	0/3	0/3
**8a**	-CH_2_C_6_H_4_ (*m*-F)	0/3	0/3	0/3	0/3
**8b**	-CH_2_C_6_H_4_ (*p*-F)	0/3	0/3	0/3	0/3
**9a**	-CH_2_C_6_H_4_ (*m*-F)	1/3	1/3	0/3	0/3
**9b**	-CH_2_C_6_H_4_ (*p*-F)	0/3	0/3	0/3	0/3

On the basis of the preliminary screening results, compounds **5i** and **5j** were subjected to the next phase of trials regarding the quantification of their anticonvulsant activity (indicated by ED_50_ MES) in mice.

The MES and subcutaneous pentylenetetrazole (scPTZ) seizure models represent the two most widely used animal seizure models in the search for new AEDs. The scPTZ test employs chemically-induced myoclonic seizures and allows the identification of agents raising the seizure threshold. This test is related to human generalized absence seizures [28]. Thus, the quantitative analysis of compounds **5i** and **5j**, the most potent compounds in the MES test, was studied in the scPTZ test to obtain ED_50_ PTZ. The quantitative neurotoxicity data (indicated by TD_50_) of compounds **5i** and **5j** was obtained from the rotarod test.

The results of the quantitative tests are reported in Table 4, along with the data from carbamazepine and phenytoin as positive drug control groups. The quantitative i.p. data in mice confirmed the safe and potent anticonvulsant activity of **5i** and **5j**. As shown in Table 4, both compounds showed a weaker anticonvulsant activity than the control drug carbamazepine (the ED_50_ MES was 11.8 mg/kg) in the MES seizure model. However, they showed a stronger anticonvulsant activity than valproic acid (the ED_50_ MES was 216.9 mg/kg) and better activities in the scPTZ test than all the drugs used as positive controls in this study. Especially, compound **5j** showed higher safety with lower neurotoxicity than compound **5i**, resulting in higher PI values (8.96 in the MES test and 9.30 in the scPTZ test).

**Table 4:** Quantitative Pharmacological Parameters ED_50_, TD_50_, and PI Values in Mice.

Comp.	ED_50_^a^ MES (mg/kg)	ED_50_ scPTZ ^b^ (mg/kg)	TD_50_^c^ (mg/kg)	PI ^d^	
				MES	scPTZ
**5i**	50.8 (37.0–69.8) ^e^	76.0 (65.9–87.7)	353.5 (309.3–404.1)	6.96	4.65
**5j**	54.8 (36.7–81.8)	52.8 (45.8–60.9)	491.0 (429.5–561.2)	8.96	9.30
**CBZ**	11.8 (8.5–16.4)	>100	76.1 (55.8–103.7)	6.45	<0.76
**VPA**	216.9 (207.5–226.3)	239.4 (209.2–274.1)	372.9 (356.0–389.8)	1.72	1.56

^a^ ED_50_: median effective dose affording anticonvulsant protection in 50% of animals; ^b^ scPTZ: subcutaneous pentylenetetrazole seizure test; ^c^ TD_50_: median toxic dose eliciting minimal neurological toxicity in 50% of animals; ^d^ PI: protective index (TD_50_/ED_50_); ^e^ 95% confidence intervals given in parentheses.

## 3. Experimental Procedures

### 3.1. General Information

Melting points were determined in open capillary tubes and were uncorrected. IR spectra were recorded (in KBr) on IR Prestige-21. ^1^H-NMR and ^13^C-NMR spectra were measured on an AV-300 (Bruker, Switzerland), and all chemical shifts were given in ppm relative to tetramethylsilane. Mass spectra were measured on an AXIMA CFR Plus MALDI-TOF (Shimadzu, Japan). The chemicals were purchased from Aldrich Chemical Corporation.

### 3.2. Chemistry

#### 3.2.1. Synthesis of 6-Hydroxy-2-aminobenzothiazole (**2**)

A mixture of 6-methoxy-2,3-dihydrobenzo[*d*]thiazol-2-amine (**1**) (10 g, 55.56 mmol) and 40 mL of hydrobromic acid (48% water solution) was refluxed for 20 h. The mixture was allowed to cool to room temperature and neutralized with NaOH solution to pH 7–8. The precipitate was filtered and washed with water. The filtrate was stirred with 100 mL hot water for 0.5 h and the remaining precipitate was filtered to yield a brown solid, compound **2**.

#### 3.2.2. General Procedure for the Synthesis of 6-Alkoxy-2-aminobenzothiazoles (**3a**–**m**)

A mixture of compound **2** (2 g, 12 mmol), potassium carbonate (2 g, 14.4 mmol), appropriate alkyl bromide or benzyl chloride derivatives (1.32 mmol), and a catalytic amount of benzyltriethylamine chloride (TEBA) in 50 mL acetone was heated under reflux for 18–24 h. After removing the solvent under reduced pressure, 80 mL of hot water was poured into the flask and the mixture was stirred for 0.5 h to eliminate the excess of potassium carbonate. The remaining precipitate was filtered to yield a russet solid (**3a**–**m**), which was used without further purification.

#### 3.2.3. General Procedure for the Synthesis of 2-Chloro-*N*-(6-alkoxybenzo[*d*]thiazol-2-yl)acetamide (**4a**–**m**)

6-Alkoxy-2-aminobenzothiazoles (**3a**–**m**) (20 mmol) was dissolved in 30 mL acetone and 30 mmol of chloroacetyl chloride were added under cold conditions. The reaction was stirred for 9–10 h at room temperature. Water was added to the reaction mixture after the solvent (acetone) was removed under pressure-reducing conditions. The mixture was stirred for 0.5 h and filtered to yield a crude product. It was then purified by silica gel column chromatography with dichloromethane to yield a white solid **4a**–**m**.

#### 3.2.4. General Procedure for the Synthesis of 2-((1*H*-1,2,4-Triazol-3-yl)thio)-*N*-(6-alkoxybenzo[*d*]thiazol-2-yl)acetamide (**5a**–**m**)

A mixture of 2-chloro-*N*-(6-alkoxybenzo[*d*]thiazol-2-yl)acetamide (**4a**–**m**) (5 mmol), 1,2,4-triazol-3-thiol (6 mmol), and sodium hydroxide (6 mmol) in *N*,*N*-dimethylformamide (10 mL) was stirred at room temperature for 12 h. After removing half of the solvent, 50 mL of water was added to the reaction mixture, which was then extracted with ethyl acetate (30 mL × 3). The combined layer of ethyl acetate was dried over anhydrous MgSO_4_. Evaporation of the solvent provided a crude product, which was recrystallized from dichloromethane to yield a white solid.

The yield, melting point, analytical data and spectral data of each compound are given below.

*2-((1H-1,2,4-Triazol-3-yl)thio)-N-(6-methoxybenzo[d]thiazol-2-yl)acetamide* (**5a**). White solid in 52.5%. mp: 208–209 °C. ^1^H-NMR (300 MHz, DMSO) δ 14.02 (s, 1H, triazole –NH–), 12.57 (s, 1H, –CO–NH–), 8.50 (s, 1H, triazole =CH–), 7.65 (d, *J* = 8.8 Hz, 1H, Ar–H), 7.58 (d, *J* = 2.5 Hz, 1H, Ar–H), 7.03 (dd, *J* = 8.8, 2.6 Hz, 1H, Ar–H), 4.20 (s, 2H, –S–CH_2_–), 3.80 (s, 3H, –OCH_3_). ^13^C-NMR (75 MHz, DMSO) δ 167.44, 156.18, 155.77, 145.31, 144.89, 142.60, 132.79, 121.21, 114.97, 104.71, 55.61, 35.07. IR (KBr) cm^−1^: 1605.02, 1556.65 (C=N). Tof-MS: *m*/*z* [M + H]^+^ 322.26.

*2-((1H-1,2,4-Triazol-3-yl)thio)-N-(6-propoxybenzo[d]thiazol-2-yl)acetamide* (**5b**). White solid in 65.9%. mp: 169–170 °C. ^1^H-NMR (300 MHz, DMSO) δ 14.09 (s, 1H, triazole –NH–), 12.50 (s, 1H, –CO–NH–), 8.51 (s, 1H, triazole =CH–), 7.64 (d, *J* = 8.8 Hz, 1H, Ar–H), 7.55 (s, 1H, Ar-H), 7.03 (dd, *J* = 8.8, 2.5 Hz, 1H, Ar–H), 4.19 (s, 2H, –S–CH_2_–), 3.97 (t, *J* = 6.4 Hz, 2H, –OCH_2_–), 1.75 (dd, *J* = 13.9, 6.8 Hz, 2H, –CH_2_–), 0.99 (t, *J* = 7.3 Hz, 3H, –CH_3_). ^13^C-NMR (75 MHz, DMSO) δ 167.41, 155.73, 155.57, 145.55, 145.23, 142.52, 132.77, 121.19, 115.33, 105.41, 69.54, 35.04, 22.09, 10.43. IR (KBr) cm^−1^: 1605.75, 1550.35 (C=N). Tof-MS: *m*/*z* [M + H]^+^ 350.15.

*2-((1H-1,2,4-Triazol-3-yl)thio)-N-(6-butoxybenzo[d]thiazol-2-yl)acetamide* (**5c**). White solid in 40.6%. mp: 172–173 °C. ^1^H-NMR (300 MHz, DMSO) δ 14.11 (s, 1H, triazole –NH–), 12.45 (s, 1H, –CO–NH–), 8.58 (s, 1H, triazole =CH–), 7.63 (d, *J* = 8.8 Hz, 1H, Ar–H), 7.55 (s, 1H, Ar–H), 7.02 (dd, *J* = 8.8, 2.5 Hz, 1H, Ar–H), 4.18 (s, 2H, –S–CH_2_–), 4.01 (t, *J* = 6.4 Hz, 2H, –OCH_2_–), 1.80–1.64 (m, 2H, –CH_2_–), 1.54–1.36 (m, 2H, –CH_2_–), 0.94 (t, *J* = 7.3 Hz, 3H, –CH_3_). ^13^C-NMR (75 MHz, DMSO) δ 167.39, 155.73, 155.64, 145.41, 145.13, 142.57, 132.81, 121.18, 115.35, 105.50, 67.84, 35.12, 30.81, 18.77, 13.67. IR (KBr) cm^−1^: 1605.78, 1556.09 (C=N). Tof-MS: *m*/*z* [M + H]^+^ 363.74.

*2-((1H-1,2,4-Triazol-3-yl)thio)-N-(6-(pentyloxy)benzo[d]thiazol-2-yl)acetamide* (**5d**). White solid in 37.2%. mp: 107–108 °C. ^1^H-NMR (300 MHz, DMSO) δ 14.07 (s, 1H, triazole –NH–), 12.46 (s, 1H, –CO–NH–), 8.51 (s, 1H, triazole =CH–), 7.63 (d, *J* = 8.8 Hz, 1H, Ar–H), 7.56 (s, 1H, Ar–H), 7.02 (dd, *J* = 8.8, 2.5 Hz, 1H, Ar–H), 4.19 (s, 2H, –S–CH_2_–), 4.00 (t, *J* = 6.5 Hz, 2H, –OCH_2_–), 1.84–1.64 (m, 2H, –CH_2_–), 1.39 (dd, *J* = 15.1, 10.5 Hz, 4H, –CH_2_–), 0.90 (t, *J* = 7.0 Hz, 3H, –CH_3_). ^13^C-NMR (75 MHz, DMSO) δ 167.41, 155.72, 155.59, 145.60, 145.26, 142.51, 132.77, 121.18, 115.32, 105.38, 68.03, 35.05, 28.43, 27.75, 21.91, 13.92. IR (KBr) cm^−1^: 1606.70, 1550.71 (C=N). Tof-MS: *m*/*z* [M + H]^+^ 378.24.

*2-((1H-1,2,4-Triazol-3-yl)thio)-N-(6-(hexyloxy)benzo[d]thiazol-2-yl)acetamide* (**5e**). White solid in 51.6%. mp: 118–119 °C. ^1^H-NMR (300 MHz, DMSO) δ 14.10 (s, 1H, triazole –NH–), 12.45 (s, 1H, –CO–NH–), 8.47 (s, 1H, triazole =CH–), 7.63 (d, *J* = 8.8 Hz, 1H, Ar–H), 7.55 (d, *J* = 2.4 Hz, 1H, Ar–H), 7.01 (dd, *J* = 8.8, 2.5 Hz, 1H, Ar–H), 4.18 (s, 2H, –S–CH_2_–), 3.99 (t, *J* = 6.5 Hz, 2H, –OCH_2_–), 1.84–1.62 (m, 2H, –CH_2_–), 1.51–1.11 (m, 6H, –CH_2_–), 0.88 (t, *J* = 6.9 Hz, 3H, –CH_3_). ^13^C-NMR (75 MHz, DMSO) δ 167.41, 155.73, 155.60, 145.38, 145.16, 142.55, 132.78, 121.19, 115.33, 105.58, 68.92, 35.09, 30.50, 28.85, 28.65, 22.11, 13.66. IR (KBr) cm^−1^: 1606.09, 1552.62 (C=N). Tof-MS: *m*/*z* [M + H]^+^ 391.99.

*2-((1H-1,2,4-Triazol-3-yl)thio)-N-(6-(heptyloxy)benzo[d]thiazol-2-yl)acetamide* (**5f**). White solid in 67.7%. mp: 147–148 °C. ^1^H-NMR (300 MHz, DMSO) δ 14.11 (s, 1H, triazole –NH–), 12.45 (s, 1H, –CO–NH–), 8.57 (s, 1H, triazole =CH–), 7.63 (d, *J* = 9.1 Hz, 1H, Ar–H), 7.55 (s, 1H, Ar–H), 7.02 (dd, *J* = 8.8, 2.5 Hz, 1H, Ar–H), 4.18 (s, 2H, –S–CH_2_–), 3.99 (t, *J* = 6.5 Hz, 2H, –OCH_2_–), 1.83–1.65 (m, 2H, –CH_2_–), 1.49–1.19 (m, 8H, –CH_2_–), 0.88 (t, *J* = 7.0 Hz, 3H, –CH_3_). ^13^C-NMR (75 MHz, DMSO) δ 167.40, 155.74, 155.65, 145.08, 145.00, 142.58, 132.83, 121.18, 115.35, 105.51, 68.16, 35.14, 31.25, 28.76, 28.45, 25.52, 22.05, 13.91. IR (KBr) cm^−1^: 1606.15, 1550.45 (C=N). Tof-MS: *m*/*z* [M + H]^+^ 406.09.

*2-((1H-1,2,4-Triazol-3-yl)thio)-N-(6-(benzyloxy)benzo[d]thiazol-2-yl)acetamide* (**5g**). White solid in 49.9%. mp: 181–182 °C. ^1^H-NMR (300 MHz, DMSO) δ 14.12 (s, 1H, triazole –NH–), 12.49 (s, 1H, –CO–NH–), 8.58 (s, 1H, triazole =CH–), 7.75–7.64 (m, 2H, Ar–H), 7.48 (d, *J* = 6.8 Hz, 2H, Ar–H), 7.41 (dd, *J* = 11.3, 4.4 Hz, 2H, Ar–H), 7.34 (dd, *J* = 8.3, 5.7 Hz, 1H, Ar–H), 7.11 (dd, *J* = 8.8, 2.6 Hz, 1H, Ar–H), 5.15 (s, 2H, –OCH_2_–), 4.19 (s, 2H, –S–CH_2_–). ^13^C-NMR (75 MHz, DMSO) δ 167.96, 156.34, 155.65, 146.36, 145.36, 143.23, 140.45, 137.42, 133.15, 128.91, 128.31, 121.72, 116.01, 106.41, 70.24, 35.40. IR (KBr) cm^−1^: 1605.62, 1551.67 (C=N). Tof-MS: *m*/*z* [M + H]^+^ 398.26.

*2-((1H-1,2,4-Triazol-3-yl)thio)-N-(6-((2-fluorobenzyl)oxy)benzo[d]thiazol-2-yl)acetamide* (**5h**). White solid in 58.3%. mp: 164–165 °C. ^1^H-NMR (300 MHz, DMSO) δ 14.10 (s, 1H, triazole –NH–), 12.51 (s, 1H, –CO–NH–), 8.56 (s, 1H, triazole =CH–), 7.76–7.70 (m, 1H), 7.69–7.64 (m, 1H), 7.59 (t, *J* = 7.1 Hz, 1H, Ar–H), 7.49–7.38 (m, 1H, Ar–H), 7.32–7.21 (m, 2H, Ar–H), 7.12 (dd, *J* = 8.8, 2.5 Hz, 1H, Ar–H), 5.18 (s, 2H, –OCH_2_–), 4.20 (s, 2H, –S–CH_2_–). ^13^C-NMR (75 MHz, DMSO) δ 167.96, 160.90 (244.5 Hz), 156.48, 155.49, 146.96, 145.36, 143.41, 133.18, 131.29, 131.24, 127.62, 125.02, 121.74, 115.98 (5.3 Hz) 115.73, 106.45, 64.64, 35.47. IR (KBr) cm^−1^: 1606.04, 1552.16 (C=N). Tof-MS: *m*/*z* [M + H]^+^ 415.52.

*2-((1H-1,2,4-Triazol-3-yl)thio)-N-(6-((3-fluorobenzyl)oxy)benzo[d]thiazol-2-yl)acetamide* (**5i**). White solid in 41.6%. mp: 171–172 °C. ^1^H-NMR (300 MHz, DMSO) δ 14.11 (s, 1H, triazole –NH–), 12.39 (s, 1H, –CO–NH–), 8.52 (s, 1H, triazole =CH–), 7.75–7.60 (m, 2H, Ar–H), 7.52–7.39 (m, 1H, Ar–H), 7.36–7.24 (m, 2H, Ar–H), 7.22–7.06 (m, 2H, Ar–H), 5.18 (s, 2H, –OCH_2_–), 4.19 (s, 2H, –S–CH_2_–). ^13^C-NMR (75 MHz, DMSO) δ 167.82, 161.53 (243.75Hz), 156.39, 155.58, 146.32, 145.89, 143.62, 140.92, 133.28, 131.70, 123.25, 121.69, 115.82, 115.42 (21.5Hz), 115.24 (6.8 Hz), 106.61, 66.87, 35.52. IR (KBr) cm^−1^: 1605.21, 1553.98 (C=N). Tof-MS: *m*/*z* [M + H]^+^ 415.68.

*2-((1H-1,2,4-Triazol-3-yl)thio)-N-(6-((4-fluorobenzyl)oxy)benzo[d]thiazol-2-yl)acetamide* (**5j**). White solid in 43.2%. mp: 206–207 °C. ^1^H-NMR (300 MHz, DMSO) δ 14.09 (s, 1H, triazole –NH–), 12.43 (s, 1H, –CO–NH–), 8.46 (s, 1H, triazole =CH–), 7.77–7.61 (m, 2H, Ar–H), 7.58–7.42 (m, 2H, Ar–H), 7.33–7.17 (m, 2H, Ar–H), 7.15–7.04 (m, 1H), 5.13 (s, 2H, –OCH_2_–), 4.20 (s, 2H, –S–CH_2_–). ^13^C-NMR (75 MHz, DMSO) δ 167.62, 161.80 (242.3 Hz) 156.72, 156.15, 155.08, 146.10, 142.88, 133.19, 132.76, 130.08 (8.3 Hz), 121.24, 115.55, 115.26 (d, *J* = 21.4 Hz), 106.02, 69.13, 35.16. IR (KBr) cm^−1^: 1604.63, 1554.77 (C=N). Tof-MS: *m*/*z* [M + H]^+^ 415.57.

*2-((1H-1,2,4-Triazol-3-yl)thio)-N-(6-((2-chlorobenzyl)oxy)benzo[d]thiazol-2-yl)acetamide* (**5k**). White solid in 33.5%. mp: 198–199 °C. ^1^H-NMR (300 MHz, DMSO) δ 14.11 (s, 1H, triazole –NH–), 12.51 (s, 1H, –CO–NH–), 8.56 (s, 1H, triazole =CH–), 7.75–7.61 (m, 3H, Ar–H), 7.58–7.50 (m, 1H, Ar–H), 7.44–7.35 (m, 2H, Ar–H), 7.13 (dd, *J* = 8.8, 2.4 Hz, 1H, Ar–H), 5.20 (s, 2H, –OCH_2_–), 4.20 (s, 2H, –S–CH_2_–). ^13^C-NMR (75 MHz, DMSO) δ 167.52, 156.13, 155.06, 146.21, 144.96, 143.05, 134.27, 132.82, 132.72, 130.18, 129.87, 129.39, 127.34, 121.32, 115.48, 106.10, 67.55, 35.10. IR (KBr) cm^−1^: 1604.77, 1558.48 (C=N). Tof-MS: *m*/*z* [M + H]^+^ 431.54.

*2-((1H-1,2,4-Triazol-3-yl)thio)-N-(6-((3-chlorobenzyl)oxy)benzo[d]thiazol-2-yl)acetamide* (**5l**). White solid in 54.3%. mp: 172–173 °C. ^1^H-NMR (300 MHz, DMSO) δ 14.10 (s, 1H, triazole –NH–), 12.52 (m, 1H, –CO–NH–), 8.55 (s, 1H, triazole =CH–), 7.74–7.62 (m, 2H, Ar–H), 7.55 (s, 1H, Ar–H), 7.50–7.34 (m, 3H, Ar–H), 7.17–7.07 (m, 1H, Ar–H), 5.17 (s, 2H, –OCH_2_–), 4.20 (s, 2H, –S–CH_2_–). ^13^C-NMR (75 MHz, DMSO) δ 167.52, 156.04, 154.96, 145.53, 145.06, 142.96, 139.56, 133.17, 132.77, 130.33, 127.79, 127.40, 126.24, 121.30, 115.53, 106.09, 68.91, 35.10. IR (KBr) cm^−1^: 1605.28, 1557.79 (C=N). Tof-MS: *m*/*z* [M + H]^+^ 431.49.

*2-((1H-1,2,4-Triazol-3-yl)thio)-N-(6-((3-(trifluoromethyl)benzyl)oxy)benzo[d]thiazol-2-yl)acetamide* (**5m**). White solid in 46.8%. mp: 182–183 °C. ^1^H-NMR (300 MHz, DMSO) δ 14.15 (s, 1H, triazole –NH–), 12.54 (s, 1H, –CO–NH–), 8.53 (s, 1H, triazole =CH–), 7.90–7.77 (m, 2H, Ar–H), 7.75–7.57 (m, 4H, Ar–H), 7.21–7.09 (m, 1H, Ar–H), 5.26 (s, 2H, –OCH_2_–), 4.20 (s, 2H, –S–CH_2_–). ^13^C-NMR (75 MHz, DMSO) δ 167.51, 156.09, 154.99, 146.22, 146.20, 145.86, 144.67, 143.05, 138.58, 132.80, 131.71, 129.56, 129.06, 124.32 (dd, *J* = 36.0, 3.3 Hz). 121.31, 115.55, 106.26, 69.08, 35.14. IR (KBr) cm^−1^: 1606.27, 1556.18 (C=N). Tof-MS: *m*/*z* [M + H]^+^ 465.42.

#### 3.2.5. General Procedure for the Synthesis of *N*-(6-Alkoxybenzo[*d*]thiazol-2-yl)-2-(1*H*-imidazol-1-yl)acetamide (**6a**–**b**)

A solution of 2-chloro-*N*-(6-alkoxybenzo[*d*]thiazol-2-yl)acetamide (5 mmol), imidazole (6 mmol), and sodium hydroxide (6 mmol) in *N*,*N*-dimethylformamide (10 mL) was stirred at room temperature for 12 h. Half of the solvent was then evaporated and the solution was poured in 50 mL water and then extracted with ethyl acetate (30 mL × 3). The ethyl acetate layer was dried over anhydrous MgSO_4_. Evaporation of the solvent yielded a crude product, which was recrystallized from dichloromethane to yield a white solid.

*N-(6-((3-Fluorobenzyl)oxy)benzo[d]thiazol-2-yl)-2-(1H-imidazol-1-yl)acetamide* (**6a**). White solid in 47.1%. mp: 224–225 °C. ^1^H-NMR (300 MHz, DMSO) δ 12.55 (s, 1H, –CO–NH–), 7.72–7.62 (m, 3H, imidazole–H and Ar–H), 7.45 (dd, *J* = 14.3, 7.4 Hz, 1H, Ar–H), 7.35–7.26 (m, 2H, Ar–H), 7.24–7.08 (m, 3H, imidazole–H and Ar–H), 6.91 (s, 1H, imidazole–H), 5.17 (s, 2H, –OCH_2_–), 5.08 (s, 2H, imidazole –CH_2_–).^13^C-NMR (75 MHz, DMSO) δ 166.89, 162.56 (d, *J* = 243.5 Hz), 156.02, 155.63, 143.32, 140.38 (d, *J* = 7.5 Hz), 138.96, 133.19, 130.89 (d, *J* = 8.3 Hz), 128.58, 124.56, 121.87, 121.35, 116.08, 115.12 (d, *J* = 21.2 Hz), 114.95 (d, *J* = 22.5 Hz), 106.61, 69.46, 49.89. IR (KBr) cm^−1^: 1604.77, 1546.91 (C=N). Tof-MS: *m*/*z* [M + H]^+^ 383.05.

*N-(6-((4-Fluorobenzyl)oxy)benzo[d]thiazol-2-yl)-2-(1H-imidazol-1-yl)acetamide* (**6b**). White solid in 56.0%. mp: 246–247 °C. ^1^H-NMR (300 MHz, DMSO) δ 12.52 (s, 1H, –CO–NH–), 7.73–7.63 (m, 3H, imidazole–H and Ar–H), 7.60–7.46 (m, 2H, Ar–H), 7.27–7.19 (m, 2H, Ar–H), 7.14–7.08 (m, 1H, Ar–H), 7.04 (s, 1H, imidazole–H), 6.92 (s, 1H, imidazole–H), 5.12 (s, 2H, –OCH_2_–), 5.09 (s, 2H, imidazole –CH_2_–). ^13^C-NMR (75 MHz, DMSO) δ 167.49, 162.26 (d, *J* = 243.7 Hz), 156.18, 155.61, 143.23, 138.88, 133.63, 133.18, 130.53 (d, *J* = 8.3 Hz), 128.46, 121.77, 121.37, 116.09, 115.72 (d, *J* = 21.4 Hz), 106.51, 69.56, 48.99. IR (KBr) cm^−1^: 1604.95, 1557.26 (C=N). Tof-MS: *m*/*z* [M + H]^+^ 383.00.

#### 3.2.6. General Procedure for the Synthesis of *N*-(6-Alkoxybenzo[*d*]thiazol-2-yl)-2-(1*H*-1,2,4-triazol-1-yl)acetamide (**7a**–**b**)

2-Chloro-*N*-(6-alkoxybenzo[*d*]thiazol-2-yl)acetamide (5 mmol) and sodium 1,2,4-triazole (6 mmol) were dissolved in *N*,*N*-dimethylformamide (10 mL) and stirred for 10 h at room temperature. Half of the solvent was then evaporated, the solution was poured in 50 mL water, and extracted with ethyl acetate (30 mL × 3). The ethyl acetate layer was dried over anhydrous MgSO_4_. After removing the solvent under reduced pressure, the crude product was obtained and recrystallized from dichloromethane to yield a white solid.

*N-(6-((3-Fluorobenzyl)oxy)benzo[d]thiazol-2-yl)-2-(1H-1,2,4-triazol-1-yl)acetamide* (**7a**). White solid in 55.1%. mp: 212–213 °C. ^1^H-NMR (300 MHz, DMSO) δ 12.73 (s, 1H, –CO–NH–), 8.59 (s, 1H, triazole–H), 8.03 (s, 1H, triazole–H), 7.77–7.64 (m, 2H, Ar–H), 7.52–7.40 (m, 1H, Ar–H), 7.37–7.27 (m, 2H, Ar–H), 7.23–7.10 (m, 2H, Ar–H), 5.33 (s, 2H, triazole –CH_2_–), 5.18 (s, 2H, –OCH_2_–). ^13^C-NMR (75 MHz, DMSO) δ 166.31, 162.67 (d, *J* = 243.8 Hz), 156.09, 155.57, 152.04, 146.23, 142.99, 140.38 (d, *J* = 7.5 Hz), 133.21, 130.94 (d, *J* = 8.4 Hz), 124.07 (d, *J* = 2.6 Hz), 121.88, 116.15, 115.08 (d, *J* = 21.0 Hz), 114.77 (d, *J* = 21.8 Hz), 106.59, 69.42, 51.57. IR (KBr) cm^−1^: 1605.72, 1556.78 (C=N). Tof-MS: *m*/*z* [M + H]^+^ 383.65.

*N-(6-((4-Fluorobenzyl)oxy)benzo[d]thiazol-2-yl)-2-(1H-1,2,4-triazol-1-yl)acetamide* (**7b**). White solid in 39.5%. mp: 227–228 °C. ^1^H-NMR (300 MHz, DMSO) δ 12.72 (s, 1H, –CO–NH–), 8.59 (s, 1H, triazole–H), 8.04 (s, 1H, triazole–H), 7.69 (dd, *J* = 5.6, 3.0 Hz, 2H, Ar–H), 7.53 (dd, *J* = 8.5, 5.7 Hz, 2H, Ar–H), 7.23 (t, *J* = 8.9 Hz, 2H, Ar–H), 7.12 (dd, *J* = 8.9, 2.5 Hz, 1H, Ar–H), 5.33 (s, 2H, triazole –CH_2_–), 5.13 (s, 2H, –OCH_2_–). ^13^C-NMR (75 MHz, DMSO) δ 166.26, 162.27(d, *J* = 242.25 Hz), 155.92, 155.70, 152.04, 146.22, 143.31, 133.65, 133.23, 130.54 (d, *J* = 8.3 Hz), 121.89, 115.72 (d, *J* = 21.4 Hz), 115.58, 106.52, 69.56, 51.56. IR (KBr) cm^−1^: 1605.71, 1556.18 (C=N). Tof-MS: *m*/*z* [M + H]^+^ 383.57.

#### 3.2.7. General Procedure for the Synthesis of *N*-(6-Alkoxybenzo[*d*]thiazol-2-yl)-2-(1*H*-tetrazol-1-yl)acetamide (**8a**–**b**)

A mixture of 2-chloro-*N*-(6-alkoxybenzo[*d*]thiazol-2-yl)acetamide (5 mmol), 1*H*-tetrazole (6 mmol), and sodium hydroxide (6 mmol) in *N*,*N*-dimethylformamide (10 mL) was stirred at room temperature for 10 h. The solution was then poured in 50 mL of water and extracted with ethyl acetate (30 mL × 3). The ethyl acetate layer was dried over anhydrous MgSO_4_. Evaporation of the solvent provided a crude product, which was recrystallized from dichloromethane to obtain a white solid.

*N-(6-((3-Fluorobenzyl)oxy)benzo[d]thiazol-2-yl)-2-(1H-tetrazol-1-yl)acetamide* (**8a**). White solid in 48.3%. mp: 205–206 °C. ^1^H-NMR (300 MHz, DMSO) δ 12.86 (s, 1H, –CO–NH–), 9.45 (s, 1H, tetrazole–H), 7.76–7.65 (m, 2H, Ar–H), 7.49–7.40 (m, 1H, Ar–H), 7.32 (s, 1H, Ar–H), 7.30 (s, 1H, Ar–H), 7.21–7.11 (m, 2H, Ar–H), 5.67 (s, 2H, tetrazole–CH_2_–), 5.18 (s, 2H, –OCH_2_–). ^13^C-NMR (75 MHz, DMSO) δ 165.43, 162.66 (d, *J* = 243.6 Hz), 156.18, 155.60, 145.76, 143.13, 140.36 (d, *J* = 7.5 Hz), 133.18, 130.95 (d, *J* = 8.3 Hz), 124.10, 121.88, 116.19, 115.10 (d, *J* = 21.2 Hz), 114.78 (d, *J* = 22.1 Hz), 106.62, 69.43, 50.11. IR (KBr) cm^−1^: 1606.36, 1556.98 (C=N). Tof-MS: *m*/*z* [M + H]^+^ 382.87.

*N-(6-((4-Fluorobenzyl)oxy)benzo[d]thiazol-2-yl)-2-(1H-tetrazol-1-yl)acetamide* (**8b**). White solid in 68.4%. mp: 224–225 °C. ^1^H-NMR (300 MHz, DMSO) δ 12.86 (s, 1H, –CO–NH–), 9.46 (s, 1H, tetrazole–H), 7.69 (s, 2H, Ar–H), 7.59–7.47 (m, 2H, Ar–H), 7.23 (t, *J* = 8.6 Hz, 2H, Ar–H), 7.13 (dd, *J* = 9.0, 1.6 Hz, 1H, Ar–H), 5.68 (s, 2H, tetrazole–CH_2_–), 5.13 (s, 2H, –OCH_2_–). ^13^C-NMR (75 MHz, DMSO) δ 166.30, 162.26 (d, *J* = 243.5Hz), 156.09, 155.58, 145.76, 143.13, 133.66, 133.23, 130.60 (d, *J* = 8.3 Hz), 121.90, 116.09, 115.69 (d, *J* = 21.2 Hz), 106.60, 69.56, 50.95. IR (KBr) cm^−1^: 1606.01, 1555.99 (C=N). Tof-MS: *m*/*z* [M + H]^+^ 382.99.

#### 3.2.8. General Procedure for the Synthesis of 2-(3-Amino-1*H*-1,2,4-triazol-1-yl)-*N*-(6-alkoxybenzo[*d*]thiazol-2-yl)acetamide (**9a**–**b**)

1*H*-1,2,4-triazol-3-amine (6 mmol), 2-chloro-*N*-(6-alkoxybenzo[*d*]thiazol-2-yl) acetamide (5 mmol), and sodium hydroxide (6 mmol) in *N*,*N*-dimethylformamide (10 mL) were dissolved and stirred at room temperature for 8 h. 50 mL of water was added to the reaction mixture, which was then extracted with ethyl acetate (30 mL × 3). The combined layer of ethyl acetate was dried over anhydrous MgSO_4_. After removing the solvent under reduced pressure, the crude product was obtained and then recrystallized from dichloromethane to yield a white solid.

*2-(3-Amino-1H-1,2,4-triazol-1-yl)-N-(6-((3-fluorobenzyl)oxy)benzo[d]thiazol-2-yl)acetamide* (**9a**). White solid in 66.1%. mp: 235–236 °C. ^1^H-NMR (300 MHz, DMSO) δ 12.58 (s, 1H, –CO–NH–), 7.70 (s, 1H, triazole–H), 7.69–7.65 (m, 1H, Ar–H), 7.50–7.41 (m, 1H, Ar–H), 7.37 (s, 1H, Ar–H), 7.34–7.26 (m, 2H, Ar–H), 7.21–7.09 (m, 2H, Ar–H), 6.33 (s, 2H, triazole–NH_2_), 5.18 (s, 2H, –OCH_2_–), 4.93 (s, 2H, triazole –CH_2_–). ^13^C-NMR (75 MHz, DMSO) δ 166.72, 162.67 (d, *J* = 243.7 Hz), 156.25, 155.49, 146.61, 145.55, 143.32, 140.39 (d, *J* = 7.4 Hz), 133.19, 130.93 (d, *J* = 8.3 Hz), 124.06 (d, *J* = 2.6 Hz), 121.80, 116.07, 115.07 (d, *J* = 21.3 Hz), 114.76 (d, *J* = 21.9 Hz), 106.57, 69.42, 49.12. IR (KBr) cm^−1^: 1604.63, 1557.39 (C=N). Tof-MS: *m*/*z* [M + H]^+^ 398.68.

*2-(3-Amino-1H-1,2,4-triazol-1-yl)-N-(6-((4-fluorobenzyl)oxy)benzo[d]thiazol-2-yl)acetamide* (**9b**). White solid in 60.2%. mp: 249–250 °C. ^1^H-NMR (300 MHz, DMSO) δ 12.51 (s, 1H, –CO–NH–), 7.72–7.64 (m, 2H, tetrazole–H and Ar–H), 7.57–7.48 (m, 2H, Ar–H), 7.37 (s, 1H, Ar–H), 7.23 (t, *J* = 8.7 Hz, 2H, Ar–H), 7.11 (d, *J* = 9.0 Hz, 1H, Ar–H), 6.34 (s, 2H, triazole–NH_2_), 5.12 (s, 2H, –OCH_2_–), 4.93 (s, 2H, triazole –CH_2_–). ^13^C-NMR (75 MHz, DMSO) δ 167.26, 162.68 (d, *J* = 243.8 Hz), 156.25, 155.58, 146.81, 145.69, 143.23, 133.68, 133.20, 130.93 (d, *J* = 8.3 Hz), 121.80, 116.08, 115.70 (d, *J* = 21.3 Hz), 106.60, 69.55, 50.18. IR (KBr) cm^−1^: 1605.88, 1556.08 (C=N). Tof-MS: *m*/*z* [M + H]^+^ 398.85.

### 3.3. Pharmacology

All compounds were evaluated for anticonvulsant activities with KunMing mice in the 18–22 g weight range purchased from the Laboratory of Animal Research, College of Pharmacy, Yanbian University. The animals were maintained on a 12 h light/dark cycle and allowed free access to food and water, except during the time they were removed from their cages for testing. The experimental substances were dissolved in dimethylsulfoxide (DMSO) and administered intraperitoneally (i.p.) in a volume of 0.1 mL/20 g body weight. The test method with reference to the Antiepileptic Drug Development (ADD) program [29,30].

#### 3.3.1. MES Screening Test

Seizures were elicited with a 60-Hz alternating current of 50 mA intensity applied via corneal electrodes for 0.2 s. Protection against the spread of MES-induced seizures was defined as the abolition of the hind leg, and tonic maximal extension component of the seizure. The MES test was performed at 30 min after compound administration.

#### 3.3.2. ScPTZ Seizures Screening Test

At 0.5 h after the administration of the test compound, 100 mg/kg PTZ (which 100% of the animals showed clonic seizure) dissolved in saline was administered subcutaneously. The animals were placed in individual cages and observed for 0.5 h. The median effective doses (ED_50_), which showed 50% protection, were recorded.

#### 3.3.3. Neurotoxicity Screening Test

The neurotoxicity of the compounds was measured in mice using the rotarod test. Mice were tested on a knurled plastic rod (diameter 3.2 cm) rotating at 6 rpm for 1 min, at 30 min after compound administration. Neurotoxicity was measured by the inability of the animal to maintain equilibrium on the rod for at least 1 minute in each of the trials.

## 4. Conclusions

In the present study, we described the synthesis and anticonvulsant activity evaluation of 2-((1*H*-1,2,4-triazol-3-yl)thio)-*N*-(6-alkoxybenzo[*d*]thiazol-2-yl) acetamide (**5a**–**m**). In addition, several series of derivatives were also synthesized and evaluated for their anticonvulsant activity for SAR studies, which included *N*-(6-alkoxybenzo[*d*]thiazol-2-yl)-2-(1*H*-imidazol-1-yl)acetamide (**6a**–**b**), *N*-(6-alkoxybenzo[*d*]thiazol-2-yl)-2-(1*H*-1,2,4-triazol-1-yl)acetamide (**7a**–**b**), *N*-(6-alkoxybenzo[*d*]thiazol2-yl)-2-(1*H*-tetrazol-1-yl)acetamide (**8a**–**b**), and 2-(3-amino-1*H*-1,2,4-triazol-1-yl)-*N*-(6-alkoxybenzo[*d*]thiazol-2-yl)acetamide (**9a**–**b**). All the compounds were tested for their anticonvulsant activity and neurotoxicity using the MES and rotarod tests at the dose of 100 mg/kg after i.p. Bioevaluation demonstrated that the compounds possessing 1,2,4-triazole-3-thiol displayed the best anticonvulsant activity and favorable PI. Among them, compounds **5i** and **5j** were the most potent, with an ED_50_ value of 50.8 mg/kg and 54.8 mg/kg in the MES test and 76.0 mg/kg and 52.8 mg/kg in the scPTZ seizures test, respectively. They also presented markedly decreased neurotoxicity and, therefore, a higher protective index. Especially, compound **5j** showed high PI values of 8.96 in the MES test and 9.30 in the scPTZ test, which were better than the standard drugs, carbamazepine and valproic acid, used as positive controls in this study.
